# The Antagonism Hypothesis: A New View on the Emergence of Consciousness

**DOI:** 10.1002/brb3.70201

**Published:** 2024-12-22

**Authors:** Weirui Xiong, Lu Yu

**Affiliations:** ^1^ School of Educational Science Chongqing Normal University Chongqing China

**Keywords:** antagonism, consciousness, first‐person perception, neuroscience, thermodynamics

## Abstract

**Purpose:**

The generation of consciousness poses a complex scientific challenge. Neuroscience and biological sciences have extensively studied this phenomenon, yielding numerous theories and hypotheses. However, to date, no reliable evidence has emerged to exclude any hypothesis conclusively, nor has any theory garnered unanimous agreement. This study aims to offer novel insights for further in‐depth study on consciousness.

**Method:**

A new theoretical hypothesis was proposed based on reviews and comments from predictive processing theory, information theory, thermodynamics, and neuroscience.

**Findings:**

This study argues that, first, it is necessary to clarify that the core implication of the concept of consciousness is first‐person perception. Accordingly, the study of consciousness is based on this premise. Second, on this basis, the antagonistic hypothesis of consciousness generation was proposed. This hypothesis holds that consciousness arises from the antagonism of mature individual experiences that cannot be seamlessly integrated with the function of addressing and navigating these conflicts.

**Conclusion:**

The antagonism hypothesis is a new concept regarding the generation of consciousness that deserves further study.

## Graphical abstract

The condition for the emergence of consciousness is antagonism, while its function is to manage and navigate antagonistic situations. Antagonism refers to obstacles that hinder the current existence of the subject or disrupt existing empirical patterns. Therefore, individuals become conscious when their current state of existence or established empirical patterns is hindered.

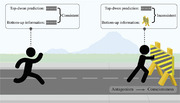

## 1 Introduction

The nature and origin of consciousness remain a major scientific problem (Reber et al. 2023). In recent years, research on the biological and physical foundations of consciousness has flourished, leading to the emergence of numerous influential theories on its nature and the circumstances surrounding its emergence. Much of this theoretical discourse revolves around the essence of consciousness and conditions conducive to its manifestation. Seth and Bayne (2022) and Sattin et al. (2021) have provided a fruitful review of the theories of consciousness that have emerged in recent years. Signorelli, Szczotka, and Prentner (2021) proposed a framework for the interpretation and classification of these theories and found that numerous competing frameworks are built on different philosophical grounds and favor particular scientific methodologies.

Contemporary psychology, which is deeply influenced by evolution, widely regards consciousness as an advanced psychological function based on the neurophysiological system. For example, higher order theories (HOT), which have received widespread attention in recent years, believe that consciousness is a meta‐characterization dependent on lower order psychological states (Rosenthal 2005) or a meta‐cognition of a general model of perceived content (Fleming 2020). Whether viewed as meta‐characterization or metacognition, consciousness is perceived as an advanced psychological function. Additionally, the brain and nervous system have long been regarded as the main carriers of advanced psychological functions, such as consciousness, making neuroscience a focal point of attention in recent years. Therefore, much of the latest progress on the origin of consciousness comes from the study of the “neural correlates of consciousness” (NCCs), which refer to a set of neural mechanisms necessary and sufficient for the emergence of consciousness (Baars 1988; Chalmers 2000; Crick and Koch 1990; Neveu et al. 2023). The purpose is to identify the brain states and processes that are closely related to consciousness (Koch et al. 2016; Seth and Bayne 2022). Sattin et al. (2021) conducted a systematic review of consciousness research papers published between 2007 and 2017, classifying and analyzing the 29 consciousness theories mentioned therein. Among the dimensions analyzed, the NCC was the most frequently reported in the full texts collected, and they found a very heterogeneous set of NCCs, ranging from a single electron to the whole brain. For example, Global Workspace Theories (GWTs) have emerged as prominent research outcomes within this framework. The core claim of the GWTs is that conscious experience is characterized by the widespread accessibility of information to consumer cognitive systems, with the frontoparietal cortex assuming a central role (Dehaene and Changeux 2011; Mashour et al. 2020).

However, the concept of NCCs has several limitations. For example, it remains challenging to determine whether the state and processes of the brain are prerequisites for consciousness or results of consciousness (Seth and Bayne 2022). The NCC framework is indispensable for consciousness science because the material foundation is also an extremely important part of consciousness research. However, despite its significance, current NCC research is far from sufficient for understanding the nature and generation of consciousness. The vigorous development of contemporary neuroscience and biological sciences has not provided reliable evidence to dismiss any particular theory. Despite the inherent complexity of consciousness, the study of consciousness in contemporary science is still in its infancy.

Simultaneously, other theories aimed at explaining how consciousness arises have also received widespread attention. For example, Balduzzi and Tononi (2008) believed that consciousness is related to a system's capacity to generate integrated information. O'Doherty (2012) argued that consciousness represents the storage of past events for use in future situations, is altered by an organism's external experience, and is a phenomenon resulting from interactions between organisms rather than being located within an organism. Additionally, the adaptive resonance theory (ART) suggests that the matching between bottom‐up external input and the top‐down expectation results in resonant states. In this framework, all conscious states are resonant states, but the reverse is not true (Grossberg 2017). Similarly, the recurrent processing theory holds that conscious visual perception requires a loop of information flow from higher order cognitive areas to lower order sensory processing areas—top‐down signaling—and the other way around—bottom‐up signaling (Lenharo 2024). This theory also suggests a relationship between top‐down signaling and bottom‐up signaling. As Hohwy (2013) stated, the predictive processing theory (PPT) also posits that consciousness originates from the interplay between top‐down predictions and bottom‐up sensory signals. Therefore, the generation of consciousness always seems to be related to both bottom‐up and top‐down processes. However, what do these two processes mean? What kind of interaction can lead to the emergence of consciousness? There are still many questions. Based on these theories and the review of consciousness research in the fields of information theory, thermodynamics, and neuroscience, this study proposes the antagonism hypothesis of consciousness, aiming to provide a fresh perspective for research in this field.

## 2 What is Consciousness?

Consciousness is both simple and complex. It is simple because nearly everyone can intuitively grasp its existence; however, it is complex because articulating its nature proves challenging. The concept of consciousness varies according to perspective. Some researchers believe that this concept refers to the specific content of consciousness, including what a person sees, smells, or hears (Zeki 2003), while others view consciousness as an individual's overall state, ranging from coma to wakefulness (Laureys and Schiff 2012). Some attribute consciousness to the pure consciousness of the self and the surrounding environment, that is, a person's alertness and ability to experience environmental/physical stimulation (Stefanelli 2023).

The different definitions of consciousness have led to different research paradigms. For example, if consciousness is regarded as an entity corresponding to matter, consciousness studies focus on describing its form, characteristics, and dynamics. However, this view is less prevalent in the modern discourse. If consciousness is regarded as an overarching state, researchers have delved into its nature, occurrence timing, and causality. For example, Chen and Chen (2022) regard consciousness as a perceptual state. Regarding consciousness as a state necessitates not only distinguishing the essential difference between conscious and unconscious states but also studying the many borderline states existing between complete consciousness and unconsciousness. These states include dreaming, sedation, nightmares, and psychedelic experiences. In a recent study, Schwitzgebel (2023) presented a strong debate on the existence of marginal consciousness. Viewing consciousness as a function or capability directs research efforts toward examining its specific roles, scope, evolutionary trajectory, and requisite conditions. Undoubtedly, consciousness represents a higher order ability than the perceptual ability. This view is welcomed by cognitive neuroscience, in which the prevailing view is that consciousness constitutes a certain function of the nervous system. For example, HOT believes that consciousness depends on the meta‐characterization of low‐level psychological states, suggesting that the generation of consciousness requires a higher level mechanism (Rosenthal 2005; Brown, Lau, and LeDoux 2019). Another example is the global neuronal workspace theory (GNWT), which asserts that consciousness depends on the ignition and propagation of a global neuronal workspace, with the frontal‐parietal cortex playing a central role (Dehaene and Changeux 2011; Mashour et al. 2020).

It is worth noting that any current definition of consciousness may be metaphorical. This is because when individuals cannot fully understand the essence of consciousness, they resort to familiar schemas from their experiences to describe and comprehend it. Much like how people struggle to imagine the shape of the universe but use familiar concepts such as eggshells to conceptualize it, consciousness may be understood through familiar scientific schemas such as particles, waves, or perceptual abilities. However, it should be noted that using a mature schema in experience can indeed help us understand consciousness more vividly, but if the nature of consciousness diverges from any known schema, it may become an obstacle to our real understanding of consciousness. Following the premise of this background knowledge, the next step is to delve into the nature of consciousness. First, it must be acknowledged that despite advancements in the science of consciousness, there remains no precise evidence to exclude any hypothesis about the nature of consciousness. Instead, these hypotheses have proliferated over time. Consciousness is a multidimensional phenomenon (Kondziella et al. 2020; Neveu et al. 2023; Walter 2021), with various definitions from different perspectives. Generally, consciousness can be defined from three perspectives: creature consciousness, state consciousness, and consciousness as an entity. The definition of consciousness in this study is based on the second perspective: consciousness as a state. Specifically, a conscious mental state is simply one in which one is aware of being (Rosenthal 1986). According to the study, consciousness is a multidimensional phenomenon related to first‐person perception. First‐person perception comes from the “first‐person problem” (Reber et al. 2023) in philosophy, which underscores the notion that the only entity one can unequivocally ascertain possesses consciousness is oneself. An individual cannot know what is in anyone else's mind. It simply points out that the only conscious entity that can be certain is an entity. Understanding another person's thoughts remains beyond one's grasp, thus emphasizing the private nature of consciousness. In this sense, consciousness embodies the states of clarity and self‐awareness. Individuals possess a distinct awareness of their existence, demonstrating a clear first‐person perception. It is important to note that consciousness is not merely a person seeing or thinking of something but a person perceiving that they are seeing or thinking of something. Therefore, HOT presents a reasonable framework. This suggests that consciousness emerges as a low‐order psychological state.

## 3 How Consciousness is Generated?

It is necessary to clarify that consciousness originates from antagonism. Antagonism in this context is not a confrontation between two entities or processes but rather a hindered process or state, similar to the concept of resistance in physics. Therefore, antagonism means that the subject's present state of being or past schema of experience is hindered and cannot run smoothly according to previous patterns. This often indicates uncertainty and error. Therefore, it is possible to assume that consciousness is prompted when individuals encounter disruptions in their automated psychological and behavioral processes. PPT posits that consciousness originates from the interplay between top‐down predictions and bottom‐up sensory signals (Hohwy 2013). Therefore, the brain has a mechanism for minimizing prediction error, aiming to reduce the discrepancy between top‐down predictions and bottom‐up information (Stefanelli 2023). Consequently, conscious mental states arise as the brain is involved in the mechanism and attempts to deduce the underlying causes of sensory stimulation (Friston 2010). One of the contributions of the PPT is to point out two processes related to consciousness: top‐down prediction and bottom‐up perception. These processes form the basis of antagonism. Consciousness arises when top‐down predictions do not coincide with bottom‐up perceptions. In other words, discrepancies or errors between the two processes trigger the onset of consciousness or prompt consciousness for intervention. As the PPT posits, the function of consciousness is to minimize the difference between top‐down prediction and bottom‐up information. In this theory, bottom‐up perception refers to an individual's perception systems, such as vision and hearing. Top‐down prediction refers to the process through which individuals anticipate or predict incoming sensory information based on past experiences. These predictions stem from the individual's accumulated knowledge and automated empirical schema, which facilitate automatic responses to specific stimuli. Therefore, when individuals encounter a stimulus, their empirical schemas automatically generate expectations for subsequent reactions, constituting top‐down predictions. However, past empirical schemas are not always effective, particularly when new stimuli fall outside the scope of the previous experience. At this time, discrepancies arise between the old empirical schema and new perceived information, leading to errors or antagonism. Therefore, consciousness arises not solely from the disparity between top‐down prediction and bottom‐up information but rather from the discrepancy between the old empirical schema and newly perceived information. Before this, the free energy principle proposed by Friston and Stephan (2007) also highlighted a similar point (Solms 2018, 2021). This principle suggests that the brain predicts the most probable experience in a given environment and compares these predictions with the actual sensory information received. If this prediction is incorrect, higher levels of the nervous system are required (Friston and Stephan 2007; Ramstead, Badcock, and Friston 2018). Solms (2018) even directly proposed that “consciousness is felt uncertainty” (Rorot 2021).

### 3.1 The Point of View of Information Theory

Let us consider the views derived from information theory. Entropy, also known as uncertainty, is an important concept in information theory that quantifies the extent of unknown information (Adami 2016). Beshkar (2023) believed that when an observer's informational uncertainty (i.e., entropy) regarding a stimulus is reduced below a critical value, the observer becomes aware of the stimulus. In simpler terms, when an observer's prior belief about a stimulus becomes entangled with the representation of that stimulus, the observer attains complete certainty about the stimulus. This complete certainty is necessary for an observer to perceive a stimulus. Thus, according to Beshkar (2023), when an observer has more uncertainty about a stimulus, consciousness is less likely to emerge; conversely, the less uncertainty an observer has, the more likely consciousness becomes. However, the opposite may also be true in practice. Therefore, greater uncertainty may increase the likelihood of consciousness. This can be found in individual experiences. Many individuals have encountered situations in life where engaging in simple, regular, uncertain, but interesting tasks makes it easy to enter a state similar to what positive psychology calls “flow” (Ren 2006). An important feature of this state is the transcendence of self‐awareness or a sense of losing oneself during the activity. However, when faced with a very uncertain problem during this continuous process, individuals often experience a sudden “awakening” state and an experience of “I'm doing something,” which is the first‐person experience. Therefore, the emergence of consciousness does not necessarily stem from a reduction in uncertainty but rather from its increase. Compared to other simpler organisms, the human living environment faces greater complexity and uncertainty. However, simpler organisms that dwell in almost entirely specific environments do not generate consciousness. In reality, the more complex the living environment, the greater the uncertainty faced by creatures and the higher the level of consciousness tends to be.

### 3.2 Thermodynamic Point of View

Regarding the relationship between consciousness and thermodynamics, it is believed that consciousness stems from computation (Dehaene, Lau, and Kouider 2017) and calculation generates heat (Beshkar 2023). In computer science, the heat generated during computing is an obstacle to circuit miniaturization (del Rio et al. 2011). Human consciousness undoubtedly involves numerous computational processes, theoretically leading to an increase in the heat of brain circuits. However, Beshkar (2023) believed that consciousness not only fails to increase but also reduces the temperature of brain circuits. When the brain produces consciousness, the temperature of the brain circuits drops. In other words, one function of consciousness is to cool the brain. Although this may initially appear contradictory, it is important to distinguish between the generation of consciousness and its function. According to Dehaene, Lau, and Kouider (2017) and others, consciousness originates from computation. However, this view is incomplete. Combining the PPT's point of view and information theory, one can see that not all computation processes are conducive to the generation of consciousness. Instead, calculating processes full of antagonism and challenges are more likely to generate consciousness. As computation generates heat, the generation of consciousness is inevitably accompanied by an increase in heat.

Will the heat continue to increase after consciousness is generated? This has to do with the function of consciousness. Beshkar (2023) believed that one function of consciousness is to cool the brain. In fact, the function of consciousness is not so much to cool the brain as to deal with antagonism or obstruction. When antagonism or obstruction is gone, the heat is reduced and the temperature is lowered. Thus, it can be said that the function of consciousness is to deal with antagonism or uncertainty. Consider the following scenario: an individual strolls along a scenic road with only one clear path devoid of uncertainty or antagonism. In this situation, the individual progresses steadily, possibly experiencing a state of “flow,” forgetting oneself. There is suddenly a fork in the road ahead. At this point, either uncertainty or antagonism occurs. The individual may suddenly awaken, immediately realize what he/she is doing, and gain a clear self‐awareness. At this time, self‐awareness is generated due to uncertainty, and the function of consciousness is to resolve this uncertainty. Once a decision is made and uncertainty is addressed, the individual's sense of uncertainty diminishes. Therefore, it can be said that consciousness arises from uncertainty or antagonism, and its function is to address and respond to these uncertainties or antagonisms. When consciousness increases, uncertainty or antagonism is reduced accordingly. From the viewpoint of thermodynamics, the logic is the same: the calculation generates heat, particularly when dealing with calculations that are full of uncertainty and difficulties. Therefore, the production process of consciousness is accompanied by an increase in heat. The function of consciousness is to manage uncertain and difficult calculations. Therefore, once consciousness is generated, these uncertain and difficult calculations will be reduced, and the temperature of the brain circuit will also decrease. Therefore, the viewpoint proposed by Beshkar (2023) suggests that consciousness that cools the brain is trustworthy.

### 3.3 The View of Neuroscience

As mentioned earlier, the latest research in this field is focused on the NCC, which constitutes a set of neurological mechanisms closely related to the emergence of consciousness (Neveu et al. 2023). Although research in this field remains controversial (Seth and Bayne 2022), NCC research remains indispensable to the study of consciousness. The latest research demonstrates that conscious activity, even in response to simple external stimuli, is possible only if the external influence and the internal dynamics of the nervous system are coordinated (Kanaev and Dryaeva 2023; Northoff and Lamme et al. 2020). The state of consciousness can be classified according to the degree of coordination between the nervous system activities and the external environment. If the internal nervous activity induced by an external stimulus is lower than normal, consciousness will not be produced, such as during sleep. Similarly, if the internal nervous system activity induced by the same external stimulus is higher than normal, consciousness is not produced, such as during hallucinations. Therefore, in general, the coordination between nervous system activities and external stimuli enables consciousness (Alu et al. 2020). However, the question arises as to whether the coordination between nervous system activities and external stimuli is a condition for the emergence of consciousness or the result of consciousness. The answer to this question is the latter. If coordination is a condition for consciousness, why would the same external stimulation induce varying degrees of internal nervous system activity? It is necessary to explain the external reasons other than the level of consciousness. However, neuroscience has not found any other significant reason for this. As Northoff (2018) advocated, the normal functioning of consciousness includes maintaining harmony between the dynamics of the nervous system itself and its openness to the external environment. Consciousness itself is closely related to incoherence. As Huang et al. (2020) stated, the unconscious state follows the simplest neural connection pathway with predictable brain dynamics, while conscious awakeness is not unpredictable and involves an inhibitory function. This result is also supported by Alu (2020); Pezzulo, Zorzi, and Corbetta (2021); and others. This conclusion can also be confirmed through everyday experiences. For example, in a hypnotic state, an individual's perceptual abilities function normally, but there is no first‐person perception. During hypnosis, individuals remain fully responsive to external stimuli (suggestives) without experiencing any antagonism or first‐person consciousness. In neuroscience, consciousness is also associated with many characteristics, such as unpredictability, uncertainty, suppression, and incongruity. This is consistent with the views of information theory and thermodynamics.

## 4 The Antagonism Hypothesis of Consciousness

As mentioned earlier, the generation of consciousness is related to uncertainty, unpredictability, a lack of coordination, and antagonism. Generally, the lower the level of certainty and predictability of their activities, the higher the level of uncertainty and unpredictability. Naturally, higher level creatures tend to exhibit higher levels of consciousness. Questions arise regarding the meaning of uncertainty, unpredictability, lack of coordination, and antagonism. According to PPT, antagonism comes from the difference or incongruity between top‐down predictions and bottom‐up perception (Friston 2010; Stefanelli 2023). From an information theory perspective, antagonism arises from an observer's uncertainty regarding the stimuli (Beshkar 2023). In thermodynamics, antagonism denotes a more challenging computational process, whereas neuroscience views it as an incongruity between internal nervous system activity and external stimuli. The PPT does not explain the top‐down prediction process in detail. This study believes that this process originates from a mature empirical schema formed through past experiences that anticipate newly perceived information. This mature empirical schema may be conditioned behaviors or automated thinking diagrams. Essentially, consciousness arises when new stimuli do not align with or are inconsistent with existing empirical schemas. Similarly, the observer's uncertainty in information theory implies the observer's inability to explain new stimuli based on inherent experiences or to incorporate them into a fixed framework. In thermodynamics, a challenging calculation process often refers to problems that cannot be solved using previous methods, whereas a simple calculation process often represents a familiar and already experienced calculation process. Neuroscience follows the same logic, suggesting that incongruity occurs when external stimuli induce new neurological activities that differ from those of past experiences. Therefore, there is reason to believe that consciousness arises from antagonism and that mature experience schema cannot match new stimuli and information. However, this statement lacks rigor and does not align with reality. Another interpretation may be more in line with the fact that, when faced with new stimuli, the original mature experience schema cannot run smoothly. This is because new stimuli do not match any experience schema but can match multiple schemas simultaneously, with each having an equal probability of induction. This can lead to the emergence of consciousness. Consider situations in which individuals exhibit heightened consciousness in dilemmas; here, it is not that mature empirical schemas do not align with new stimuli but rather that they cannot function smoothly. Hence, this study adopts the concepts of antagonism over uncertainty, incongruity, unpredictability, and other concepts.

As mentioned previously, it is necessary to separate the conditions for the generation of consciousness from the function of consciousness. According to this study, consciousness arises from antagonism, and its function is to address this antagonism. As Northoff (2018) advocated, the normal function of consciousness includes maintaining harmony between the dynamics of the nervous system and its receptivity to the external environment. In other words, consciousness arises from antagonism caused by the inability of a mature empirical schema to function smoothly, and the process of consciousness is to eliminate this antagonism, such as by generating a new empirical schema and enabling it to run smoothly. Therefore, consciousness is not only a sense of uncertainty (Rorot 2021) but also an ability or process to resolve uncertainty. Consciousness is generated in the process of solving challenging problems and can aid individuals in problem‐solving. This explains why individuals often forget their existence when engaged in simple tasks and suddenly feel awake when faced with difficult problems. The fact that consciousness consumes energy is inevitable, and as posited by information theory and thermodynamics, consciousness comes from calculation, which generates heat (Beshkar 2023). There is reason to believe that the more challenging the calculation, the more energy it consumes and the more heat it generates. Many people have experienced that the simpler a task, the easier it is, whereas solving more difficult tasks leads to a clearer state of consciousness and increased fatigue. Negative emotions such as anxiety often accompany problem‐solving situations. People may perceive time to pass slower during such times because their level of consciousness is heightened, resulting in a clearer first‐person perception. In situations characterized by positive emotions, where things often go smoothly, the level of consciousness awakening is low, and there is no clear first‐person perception. Thus, the passage of time occurs more rapidly.

To clarify this theory, we examine some relevant examples. The latest study by Steinmassl and Paulus (2024) mentioned the enfacement illusion, in which the participant is stroked by a cotton bud on the cheek while watching another person being stroked by an identical cotton bud on a congruent spot (interpersonal multisensory stimulation [IMS]). If stroking is executed synchronously, multisensory integration processes lead to the experience of ownership over the other's face and increased self–other overlap or self–other blurring (Tsakiris 2008). As Gallup (1970) stated, distinguishing between one's own face and that of others is considered an essential part of the body's self‐awareness. Therefore, when the subjects experienced an enfacement illusion, there is no clear physical self‐awareness. This is because when the subjects are watching other people's cheeks being stroked by the cotton bud, they will generate a top‐down expectation, and if, at the same time, the cotton bud is also touching their own cheeks in the same way, the bottom‐up information is consistent with top‐down expectations, or the top‐down expectations are not hindered or antagonized, so there will be no clear physical self‐awareness. Once the frequency of being touched by a cotton bud on one's cheek is inconsistent with the frequency of being touched on the cheek of others in the video, there will be an inconsistency between the bottom‐up information and the top‐down expectation, that is, the top‐down expectation is antagonized, and the subjects immediately become conscious of their bodies. It is conceivable that if the subject is always in a state of synchronous sensory stimulation, he is likely to always be in a state of self‐other overlap or self‐other blurring. Therefore, as claimed by the antagonism hypothesis, the emergence of consciousness is always associated with antagonistic processes. Another example is the sleep phenomenon, which is regarded as a valuable window to understand consciousness. From the perspective of antagonism, whether it is sleep or hypnosis, the subject is required to remove various physical and psychological antagonisms through relaxation so that it is easy to achieve an unconscious sleep or hypnosis state. Once an external force is applied to break this unhindered state (i.e., to be antagonized), people will wake up. Tononi, Boly, and Cirelli (2024) found that regardless of the behavioral state, consciousness exists as long as the neural substrate is not in a state of bistability. In addition, many automated behaviors are always associated with unconsciousness, as Knafo and Weinberger (2024) pointed out in their study of hypnosis. In fact, the meaning of automated behavior itself is that a certain behavior will not be hindered and can run smoothly. Once certain factors hinder this automated behavior, that is, antagonism, it is easily noticed by people and enters a conscious state. For example, the “FLOW” phenomenon mentioned in positive psychology can also illustrate this point. When people devote themselves to some simple work, after a period of time, they will forget themselves and experience unconscious FLOW. This is because the work that people do is simple and can be performed automatically based on their experience. Once a difficult problem arises, this automated working mode is antagonistic so that the individual seems to “wake up” in an instant, thus having consciousness. As Weinberger and Stoycheva (2020) argued, nothing is purely conscious or purely unconscious, and there is no clear boundary to distinguish between the two. Therefore, it is not easy to completely and accurately distinguish which processes are conscious from which processes are unconscious through the antagonistic hypothesis or to find the critical value to distinguish between conscious and unconscious. This hypothesis is only a preliminary hypothesis or direction for research on the generation of consciousness. Further research is required to explore the specific mechanism of antagonism, especially its neurophysiological basis.

## 5 Conclusion

It can be seen from the above that the concept of consciousness is consistently intertwined with first‐person perception. Only when there is clear evidence of a first‐person perception can a genuinely conscious experience be considered. When discussing the emergence of consciousness, it is important to distinguish between the conditions necessary for its emergence and its functional role. Based on a systematic review of information theory, thermodynamics, and neuroscience, this study proposes an antagonistic theory of consciousness. According to this theory, one of the necessary conditions for the emergence of consciousness is antagonism, the function of which is to manage and navigate antagonistic situations. In this context, antagonism refers to obstacles hindering the current existence of a subject or disrupting existing empirical patterns. Therefore, individuals become conscious when their current state of existence or established empirical patterns are hindered. This theoretical hypothesis is still under development, and much work needs to be done to improve it. First, antagonism is a necessary but insufficient condition for generating phenomenal consciousness. Our future research will explore other conditions for the generation of consciousness. For example, although consciousness is not equivalent to perception, the subject does indeed generate a clear “first‐person” perception at the moment of consciousness generation, so having autonomous perceptual capabilities seems to be one of the necessary conditions for the generation of phenomenal consciousness. This may explain why some physical systems that lack the neural systems associated with perceptual capabilities do not generate phenomenal consciousness, even when they exhibit antagonism. An interesting question is whether a physical system that possesses perceptual capabilities would also generate self‐consciousness when it exhibits antagonism. Additionally, when animals with a certain degree of perceptual capability exhibit antagonism, is self‐consciousness the same as human self‐consciousness? These questions warrant further investigation.

## Author Contributions

**Weirui Xiong**: conceptualization, methodology, formal analysis, writing–review and editing, writing–original draft. **Lu Yu**: writing–review and editing.

## Ethics Statement

The authors have nothing to report.

## Conflicts of Interest

The authors declare no conflicts of interest.

### Peer Review

The peer review history for this article is available at https://publons.com/publon/10.1002/brb3.70201.

## Data Availability

The authors have nothing to report.
